# Induced Transient Immune Tolerance in Ticks and Vertebrate Host: A Keystone of Tick-Borne Diseases?

**DOI:** 10.3389/fimmu.2021.625993

**Published:** 2021-02-12

**Authors:** Nathalie Boulanger, Stephen Wikel

**Affiliations:** ^1^Fédération de Médecine Translationnelle – UR7290, Early Bacterial Virulence, Group Borrelia, Université de Strasbourg, Strasbourg, France; ^2^Centre National de Référence Borrelia, Centre Hospitalier Universitaire, Strasbourg, France; ^3^Department of Medical Sciences, Frank H. Netter, M.D., School of Medicine, Quinnipiac University, Hamden, CT, United States

**Keywords:** tick, skin immunity and microbiome, immune tolerance, tick-borne diseases, innate immunity, adaptive immunity

## Abstract

Ticks and tick transmitted infectious agents are increasing global public health threats due to increasing abundance, expanding geographic ranges of vectors and pathogens, and emerging tick-borne infectious agents. Greater understanding of tick, host, and pathogen interactions will contribute to development of novel tick control and disease prevention strategies. Tick-borne pathogens adapt in multiple ways to very different tick and vertebrate host environments and defenses. Ticks effectively pharmacomodulate by its saliva host innate and adaptive immune defenses. In this review, we examine the idea that successful synergy between tick and tick-borne pathogen results in host immune tolerance that facilitates successful tick infection and feeding, creates a favorable site for pathogen introduction, modulates cutaneous and systemic immune defenses to establish infection, and contributes to successful long-term infection. Tick, host, and pathogen elements examined here include interaction of tick innate immunity and microbiome with tick-borne pathogens; tick modulation of host cutaneous defenses prior to pathogen transmission; how tick and pathogen target vertebrate host defenses that lead to different modes of interaction and host infection status (reservoir, incompetent, resistant, clinically ill); tick saliva bioactive molecules as important factors in determining those pathogens for which the tick is a competent vector; and, the need for translational studies to advance this field of study. Gaps in our understanding of these relationships are identified, that if successfully addressed, can advance the development of strategies to successfully disrupt both tick feeding and pathogen transmission.

## Introduction

**Figure 1:**
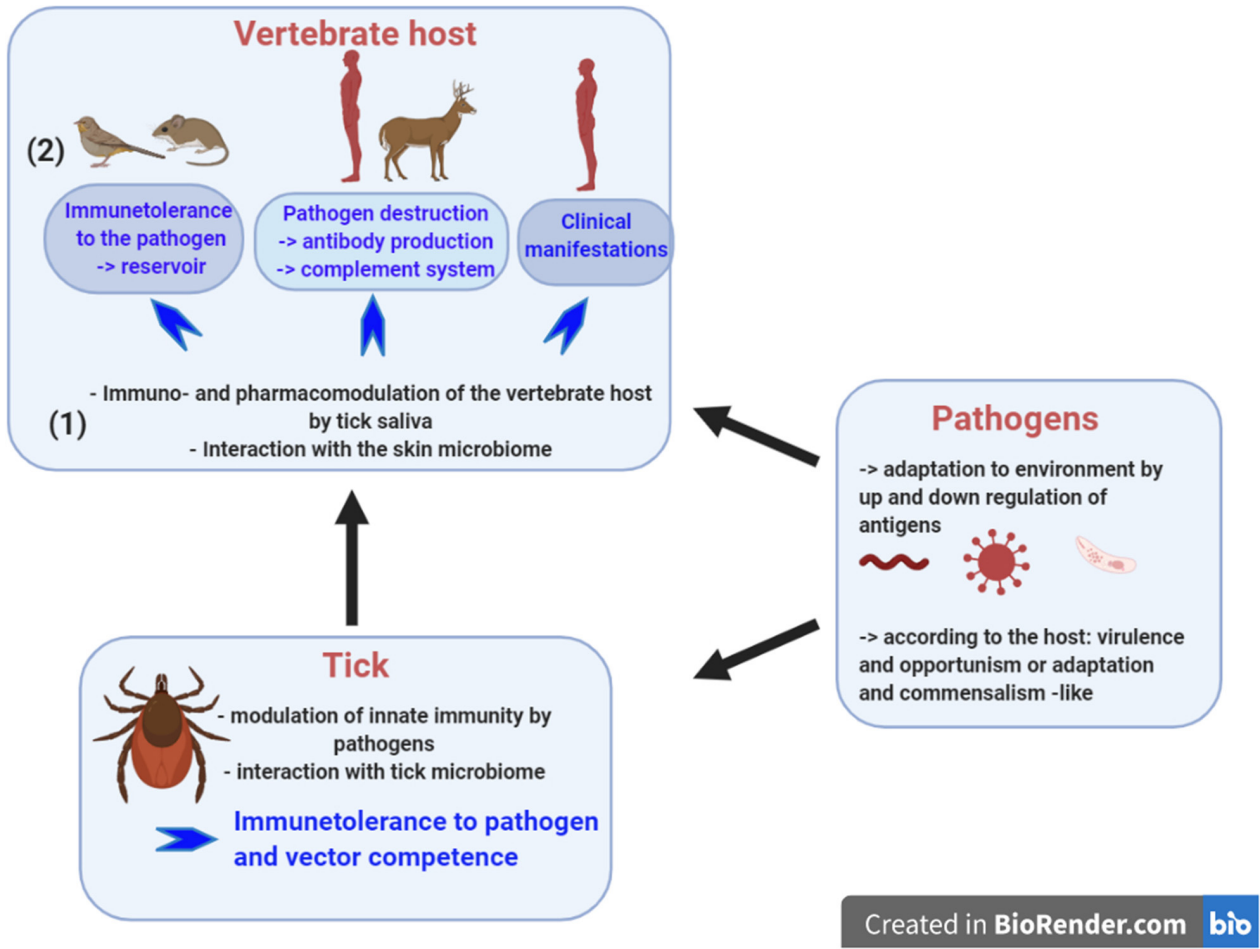
Tick-borne diseases rely on interplays between the tick, the pathogen and the vertebrate host. To be a competent vector, the tick must control the pathogen population by its innate immunity and the tick microbiome seems to contribute to this control. During pathogen inoculation into the skin, tick saliva modulates the pharmacology and the immunology of the vertebrate host. Skin immunity plays a major role in tolerance of tick-borne pathogens. It is likely that the skin microbiome participates in this immunomodulation. Once inoculated, the infection outcome varies: (1) in animal reservoir like rodents, where no clinical manifestations develop and the pathogens survive for months allowing their persistence in the environment; (2) the vertebrate host has a sufficient immune system to neutralize the pathogens and antibody presence provides evidence of contact with the pathogens; and, (3) the vertebrate host does not trigger a sufficient and protective immune response and as a consequence develops clinical disease. Created with BioRender.com.

Tick-borne diseases initially viewed as a triad of vector-pathogen-host, have evolved toward a very complex network of interactions. A fourth actor has appeared, the microbiome, present within the tick (1, 2), but also at the skin interface of the vertebrate host (3) (Figure 1). More recently, a fourth factor has emerged as an important cellular regulator, the non-coding RNAs (4).

Tick-borne pathogens should be viewed as danger signals, a concept developed by Polly Matzinger in 1994 (5, 6) and later refined by Medzhitov and Janeway (7). How do these pathogens manipulate the tick and the vertebrate host immunity to not be eliminated? Up and down regulation of antigens helps the pathogens to adapt to its environment. Significantly, the tick itself must also be considered as a danger signal for the vertebrate host during the bite process, however its saliva makes it tolerant for the immune system of the vertebrate host. Tick modulation complements the contribution of tick-borne pathogen manipulation of the host environment (Figure 1). Tick saliva prepares the site of inoculation and makes it tolerant for inoculated pathogens, except for viruses that are inoculated within a few minutes of starting the blood meal. A delay of 12–24 h or more in pathogen inoculation is observed for bacteria and parasites, transmitted by hard ticks (8, 9). The final outcome of this tripartite relationship is determined by the interplay of the immune responses of the host and tick vector on the pathogen; modulation of vertebrate host defenses by the tick and pathogen; and the largely unknown manipulation of tick innate immunity by the tick transmitted pathogen.

Major advances in immunology will help to understand the different levels of interactions and tolerance which occur in tick-borne diseases. What are the role of the different T cell populations such as the Treg or the T_RM_ (T resident memory cells) (10) and Innate Lymphoid Cells (11) in the control of infection at the skin interface? Skin immunity should be particularly investigated since the skin represents a site of pathogen inoculation, and for some tick-borne pathogens a site of multiplication and persistence. For example, why does *Borrelia burgdorferi* sensu lato (sl), the bacteria responsible for Lyme borreliosis, multiply so intensively in the skin early after its inoculation (12)? Does it take advantage of the immunologically permissive environment created by tick modulation of host defenses? Is it to induce an immune tolerance and facilitate *Borrelia* persistence in the skin for months (13)? Additional factors might help successful tick-borne multiplication and persistence. While the role of adipocytes and hair follicle has been shown for *Plasmodium* in malaria infection (14, 15) and for *Trypanosoma* in sleeping sickness (15, 16), for tick-borne diseases these relationships are yet to be defined.

New technologies should help to answer some of these questions. They have greatly evolved from early proteomics and transcriptomics to more powerful functional genomic, deep sequencing and bioinformatics analyses (17). With single cell technology, we might expect to unravel the complex interactions of host-pathogen-tick interaction (18). In this review, we will present the gaps existing presently to understand the different interactions taking place during the complex travel of tick-borne pathogens through the vector and the vertebrate host. We will also highlight some recent advances in skin immunity and its microbiome that we should explore.

## Tick

Ticks are an ancient group of organisms that transmit a large array of pathogens, more than other haematophagous arthropods. This is likely explained by their life cycle, spending their free life in leaf litter and humus rich in microorganisms and then as an ectoparasite on vertebrate host skin rich in other types of microorganisms, microbiota (3), that can be potentially acquired during the course of their long blood meal. To adapt to these different environments, ticks developed innate immunity (19). Some of these tick associated microorganisms are endosymbionts and others evolved to become tick-transmitted pathogens that are responsible for tick-borne diseases (2). Tick-borne pathogens possibly circumvent or actively modulate tick innate immune defenses, resulting in tolerance to their presence within the tick vector.

### Tick Innate Immunity

To defend itself from microbial insults and injury, ticks rely solely on innate immunity. Microbial insults can be generated through their blood meal or in response to physical damage to the cuticle. Tick immune system comprises central tissues like fat body, the equivalent of vertebrate liver and adipose tissue, and different types of hemocytes. In the periphery, the epithelium of different organs secretes effector molecules to protect ticks (20). This innate immune system can be particularly challenged during the blood meal. Ticks are strictly hematophagous, and all events occurring during the blood meal can induce the immune system, especially if the tick feeds on an infected vertebrate host.

The innate immunity of the tick relies on different structures. Mesodermic **fat body** is present in all tick stages. It is located beneath the epidermis and around organs, particularly the trachea. It is mainly a source of vitellogenin, but also a source of antimicrobial molecules secreted into the hemolymph (21). In the **hemolymph**, tick innate immunity relies on cellular immunity including active phagocytosis, nodulation and encapsulation orchestrated by **hemocytes** circulating in an open circulatory system. In ixodid ticks, three types of hemocytes have been described: prohemocytes, granulocytes and plasmatocytes that participate in phagocytosis, clotting system, and encapsulation of microbes (22). More recently, humoral immunity has been investigated in ticks, building on research on *Drosophila melanogaster* (23). The discovery of cecropin, the first antimicrobial peptide (AMP) in primitive insect, *Hyalophora cecropia* (24), open the avenues to the discovery of innate immunity in *Drosophila*. The two main pathways, Toll activated by Gram-positive bacteria or fungi, and Imd activated by Gram-negative bacteria, were discovered (25), leading to the identification by homology to the cloning of Toll (TLR—toll-like receptors) in human (26). In addition to these specific immune organs, some barriers protect the tick. While feeding, a **peritrophic membrane** (PM) is formed by secretion from the midgut epithelium, at least in some species of Ixodidae (27). This chitin-rich matrix formation was first described in the three life stages of *I. ricinus*. It appears 18 h after the beginning of blood digestion and remains intact for several days (28). It surrounds the blood meal and protects the midgut epithelium. Then, the blood digestion occurs intracellularly *via* phagocytosis into the midgut cells (29). The epithelium is the next component of the **gut barrier** that operates upon uptake of the blood meal and movement of cells and fluid across the gut to the hemolymph. The innate immunity of the epithelium has been well-investigated in insects, particularly in *Drosophila* (30) and in the *Anopheles* mosquito as insect vector (30). This topic deserves greater examination in tick-pathogen interactions (31).

Tick innate immunity is regulated by different pathways and molecules. Hemocytes, midgut epithelium, and salivary glands produce defensive, anti-microbial molecules (31). This generally happens upon recognition of the pathogen-associated molecular patterns (PAMPs) of microorganisms by specific receptors. In *Drosophila*, some of these receptors include the peptidoglycan recognition proteins (PGRPs) and the Gram-negative binding proteins (GNBPs). In tick genome, all the components of these two cascades have not been identified so far (32, 33). Interestingly in *Ixodes* ticks, lipid moieties (1-palmitoyl-2-oleoyl-sn-glycero-3-phosphoglycerol or POPG and 1-palmitoyl-2-oleoyl diacylglycerol or PODAG) of certain pathogens elicit IMD (Immune deficiency) pathway (34). In hemolymph, activation of different proteolytic cascades is triggered similar to a complement cascade and clotting cascade, but no phenoloxidase cascade has been identified in tick (19, 35). This activation leads to release of soluble factors and antimicrobial molecules (lysozyme, defensin and hemoglobin peptides) (31). In addition to the well-known defensins, some molecules have been identified in ticks: microplusin, ixodidin and hebraein (36). Microplusin for example has a bacteriostatic effect by sequestering copper used by bacteria (19). Other soluble factors in tick humoral immunity include antimicrobial proteins such as lectins, lysozyme, proteases and inhibitors of proteases like alpha2-macroglobulin. This molecule belongs to the thioester-containing proteins with similarity to the C3-components of the complement system and insect TEPs that inhibit pathogen proteases.

Regulation of the tick innate immune response relies on three pathways: IMD, JAK-STAT and Toll pathways. Activation occurs through different mechanisms of pathogen recognition and leads to secretion of effector molecules that further neutralize the pathogens. The IMD pathway has been the most investigated. PAMP recognition leads to the interaction of XIAP (ubiquitin ligase: X-linked inhibitor of apoptosis) with its substrate, the protein p47. The silencing of this protein *in vitro* and *in vivo* affects pathogen control and enhances *A. phagocytophilum* intracellular burden in ISE6 hemocytes and *B. burgdorferi* infection of *I. scapularis* nymphs (34, 37). This pathway also protects *Dermacentor andersoni* against *A. marginale* (34). In the **gut epithelium** of *I. scapularis*, the mRNA of a defensin-like peptide was identified; the amino-acid sequence having 79 % similarity with a *D. variabilis* defensin (38). In *Haemaphysalis longicornis*, an antimicrobial peptide, longicin, was also expressed in the gut and had antimicrobial activity on different microbes (bacteria, fungi, and *Babesia*) (39). In addition, the JAK/STAT pathway is a key signaling pathway in gut immunity in *I. scapularis*, as was shown in *Drosophila* (32). In tick hemolymph, defensins were identified as effector molecules as well as lectins and TEP (thioester-containing proteins) (19). Similarly, in salivary glands, transcriptomic studies revealed the presence of AMPs in different tick genera (19). By comparative genomics, an RNA interference (RNAi) pathway has also been described (40), that is, mainly involved during tick-virus interactions. This is a gene silencing process triggered upon interaction with double-stranded RNA. Most viruses infecting ticks are RNA viruses (36). In *Ixodes scapularis* tick, genome sequencing identified several genes participating in these different pathways, but characterizations of some components are still missing (19, 41). The use of tick hemocyte cell lines has been proposed to investigate the molecular mechanism involved in tick immune response. The interaction of ISE6 hemocytes with *A. phagocytophilum* has been particularly explored (34, 37, 42). More precisely, using metabolomics, transcriptomics and proteomics, it has been shown that the intracellular bacterium affects the protein processing in the endoplasmic reticulum and decreases the glucose metabolism. The bacterium also limits the tick immune response within the hemocytes and inhibits apoptosis facilitating its survival and possibly its further transmission to the vertebrate host (42). Through its co-evolution of more than 300 million years (43), it seems that pathogens establish with the tick innate immune system an intimate equilibrium at different levels, where both need to survive. The tick is not killed by the tick-borne pathogen and the pathogen population is controlled by the tick immunity limiting damage to the tick.

During the blood meal, some molecules of the vertebrate host can interact with the tick immune system. Recently, the JAK-STAT pathway has been shown to be controlled by cross species signaling between mice and ticks (44). Indeed, **mouse INF-gamma** acquired during the blood meal on a *Borrelia*-infected mouse activated *Ixodes* STAT leading to the secretion of the antimicrobial peptide (AMP) Dae2 that in turn controls the *Borrelia* population in the gut of the infected tick. Similarly, this pathway was shown to regulate the *A. phagocytophilum* population in ticks (45). Although different components of the Toll pathway were identified in ticks, its direct involvement in pathogen control was not yet demonstrated. Host **hemoglobin** also participates in the control of infection against Gram (+) bacteria and fungi. In the tick midgut, hemoglobin is cleaved in large peptides (hemocidins) with antimicrobial activity. A seminal observation was that hemoglobin passed across the gut from the blood meal of the insect, *Rhodinus prolixus*, into hemolymph and subsequently was incorporated into salivary glands (46). This phenomenon has been observed in both soft and hard ticks (19). Host-derived **plasminogen** also helps some pathogens to escape tick immunity and facilitates their migration through the gut epithelium, as shown for *Borrelia* (47).The outcome of **host immunoglobulin** in the tick has been particularly investigated. Immunoglobulins consumed in the tick blood meal passed serologically intact across tick gut into hemolymph and subsequently were detected in salivary gland extract (48, 49). Immunofluorescent microscopy confirmed that rabbit antibodies raised against tick ovaries and salivary glands, when consumed in a blood meal, retained tissue antigen binding specificity in *D. variabilis* hemolymph (48). Likewise, hemolysins raised in rabbits to sheep erythrocytes retained their antigen specificity in the hemolymph of female *I. ricinus*. Ticks fed upon re-infested rabbits had higher titers of hemolysin in their hemolymph than observed during an initial infestation (50). Intact rabbit immunoglobulin G was also present, post-blood meal, in hemolymph of the argasid tick, *Ornithodoros moubata* (51). Argasid ticks consume a much smaller blood meal in 2 h, while ixodid larvae and nymphs may complete a blood meal in 4 days and adults may require more than a week (52, 53).

What are the quantitative aspects of host immunoglobulin in hemolymph of argasid and ixodid species? Concentration of host immunoglobulin G was found in a comparative study of seven species to be highest in *Hyalomma excavatum* with 30% intact (54). Immunoglobulin concentration in *O. moubata* was comparatively very low; however, 100% of the molecules were intact (54). Blood meal immunoglobulin G did not pass into hemolymph of the argasid species, *Argas persicus* and *Ornithodoros tholozani* (54). Hemolymph immunoglobulin G antibody specific activity was 35 to 42% for *Rhipicephalus appendiculatus* females that fed upon guinea pigs immunized with killed *Escherichia coli* (49). Immunoglobulin binding proteins present in both hemolymph and salivary glands of *R. appendiculatus* were hypothesized to be involved in removing foreign proteins from the tick (49). Functional bovine antibodies persisted in *Rhipicephalus (Boophilus) microplus* hemolymph for at least 48 h post-engorgement (55). Host immunoglobulin G entered *Amblyomma americanum* adult hemolymph at 6.5% of the concentration in the capillary tube feeding solution after 6 h with no evidence of binding to cells (56). The Fc piece was identified as the immunoglobulin G molecule region essential for specific uptake across the *A. americanum* midgut into hemolymph with receptor mediated endocytosis speculated to be the mechanism for preferential transport of immunoglobin G from midgut to hemolymph (57).

Why is host immunoglobulin taken up from the blood meal, moved to the hemolymph, and then found in the salivary glands of a feeding tick? This process may be a means of removing large proteins in the blood meal. Could antibody molecules recycled back into the host bite site down regulate host immune defenses? Host species can be immunized with tick internal tissue molecules essential for normal physiological function that results in antibodies taken up in the blood meal moving from tick midgut into the hemolymph that bathes internal tissues where host antibodies can bind and disrupt tick physiological pathways and cell function.

### Tick Microbiome and Virome

In addition to pathogenic microorganisms, the tick also harbors symbiotic microorganisms. A few years ago, two metagenomics studies were performed to analyze the bacterial diversity of the cattle tick, *R. microplus* (58) and *I. ricinus* (59). In both studies, more than a 100 bacterial genera were identified in the different tick stages. Variations were found according to geography and environment. Among all genospecies of hard ticks, the most studied group, bacteria of the phylum *Proteobacteria* are predominant followed by *Firmicutes, Actinobacteria*, and *Bacteroidetes*. These endosymbionts likely either evolved toward virulent vertebrate pathogens or colonized ticks to become endosymbionts of specific tick tissues (1). Mechanisms that govern this evolution toward virulent microorganisms remain to be elucidated. Within the tick, these symbionts are well-known for their beneficial effects, notably their role on arthropod nutrition as a provider of B vitamin. The nutritional role of symbionts has been particularly well-investigated in the model tsetse-fly-*Trypanosoma* for a potential use in paratransgenesis (60, 61). In ticks, it has also drawn attention for the biocontrol of ticks with a first study in 1998 on *Ixodes scapularis* microbiome (62). Then, with the development of metagenomics, additional tick genera have been investigated for their microbiome and the list of identified microorganisms has been implemented (58, 59). In *Ixodes*, among the different bacterial families characterized, the Enterobacteriaceae have been shown as essential bacteria in the tick microbiome (63). An assay of vaccination with one of these bacteria, *Escherichia coli*, has been tempted in C57BL/6 mice deficient alpha-1,3-galactosyltransferase. Indeed, this sugar residue is broadly distributed in bacteria, fungi and mammals except humans and old word monkeys (63). *Ixodes* nymphs that fed on these immunized mice were hampered in their blood meal and showed high mortality. BalB/c and C57BL/6 mice were not affected, pointing out the role of the genetic background in the response to this sugar residue (63).

Three bacteria genera, *Coxiella, Francisella*, and *Rickettsia* are particularly interesting in ticks since within each of these genera, some evolved as true pathogens while others are endosymbionts (2). Some symbionts seem also to directly compete with TBP as described within the *Rickettsia* genus in *Dermacentor*. An interesting relationship operates in *Dermacentor andersoni* between non-pathogenic, *Rickettsia peacockii*, and pathogenic rickettiae, *Rickettsia rickettsii*, in tick vectors. This relationship goes back to the Bitterroot Valley of Montana with the non-pathogenic “east side agent” and highly pathogenic *Rickettsia rickettsii* on the west side of the valley (64). Interestingly, when both *Rickettsiae* are present within the tick, *R. rickettsii* has a reduced prevalence and the incidence of Rocky Mountain Spotted Fever is reduced. Genome sequence analysis of the two *Rickettsia* species revealed that the virulence could be mainly associated with an ankyrin repeat containing protein (65).

The roles of both microbiome and virome in development of tick innate immunity and immune tolerance to microbial agents within the vector are interesting and important topics to address.

### Tick Microbiome at the Skin Interface?

The co-transmission of vector microbiome and Vector-borne pathogen (VBP) has been suspected in insect. During regurgitation process as present in the transmission of *Yersinia pestis* by the flea or *Leishmania* by the sandfly, the presence of gut microbiome of the insect in the vertebrate host skin is likely. Stercoral transmission of *Trypanosoma cruzi* during the reduvid bite likely also involves gut microbiome deposit at the skin interface of the vertebrate host (66). Recent data on *Leishmania donovani* transmission confirmed this hypothesis. These parasites are co-inoculated with sandfly microbiota leading to inflammasome activation and secretion of IL-1 beta (67). In transmission of tick-borne pathogens, very few studies have been performed to elucidate the potential transmission of gut microbiota during the process of the tick bite *via* exosomes (68). Interestingly, some of these tick symbionts have been shown to be transmitted during the tick bite process, due to their presence in salivary glands. It has been shown for *Coxiella*-like endosymbiont, found in a human skin biopsy and inducing a human infection in Europe (69). Similarly, *Midichloria mitochondrii*, an intra-mitochondrial symbiont of hard tick has been detected in tick salivary glands and transmitted to vertebrate hosts as evidence by the presence of antibodies against the bacteria in humans (70) and in rabbits (71). Application of next generation sequencing and advanced bioinformatics tools at the site of the vector bite should help to build upon these preliminary data and identify tick microbiota present in the host. However, detailed studies are needed to investigate whether the inoculated microbiota play a role in initiating the immune response of the vertebrate host (66). A recent work (68) demonstrated that tick saliva of *Amblyomma maculatum* and *I. scapularis* contain exosomes. In an *in vitro* system using a keratinocyte cell line (HaCaT cells), the authors demonstrated that these exosomes delayed the wound healing process by down regulating CXCL12 and upregulating IL-8.

### Tick-Borne Pathogen Interaction With Tick Gut and Salivary Glands

The midgut is the largest organ with several diverticuli in the body cavity of the tick. Its size greatly enlarges during the blood meal. The digestion of blood occurs intracellularly and the midgut cells serve as a storage cells for the blood nutrient, enabling ticks to survive extended period (72). Pathogens entering the gut during the blood meal first have to overcome the acellular barrier constituted by the **peritrophic membrane** (PM). It protects the gut epithelium from injury potentially induced by ingested particulates or pathogens during the blood meal. The JAK-STAT pathway regulates its formation. Indeed, a decrease in the expression of the transcription factor STAT induces a lower expression of peritrophin, a glycoprotein of the PM (73). STAT expression is itself regulated by the gut microbiota as shown in dysbiosed larvae of *I. scapularis* (73). In this study, it is also demonstrated that the integrity of the PM is necessary for the colonization of the gut by *B. burgdorferi*. The role of a protein present in the PM, a chitin deacetylase, has been investigated in relation to *Borrelia* infection within the gut, but no clear role of the protein has been established (74). For *Anaplasma*, the opposite effect of peritrophic membrane is observed (75). The symbiotic bacteria induce the tick to synthesize a glycoprotein, IAFGP (*Ixodes scapularis* anti-freeze glycoprotein), that modifies the formation of bacteria biofilm essential for the formation of the peritrophic membrane. *A. phagocytophilum* can then more easily invade the gut tick cells and migrate to the salivary glands (75). The role of PM has also been studied for the parasite *Babesia microti*. *Babesia* goes through the PM by help of a specific parasite structure, the arrowhead (28). Finally, the precise role of PM in the context of tick-borne pathogens remains to be investigated.

Then, microorganisms need to pass through the **gut epithelium**. Unlike in insects, microorganisms ingested during the tick blood meal do not face directly the digestive enzymes in the gut lumen. Some, like *Rickettsia*, are internalized and escape endosomes and digestion, to develop in tick cell cytoplasm (36). Using a yeast surface display library of tick gut proteins, four *B. burgdorferi*-interacting tick proteins have been identified. Two have been characterized. A fibronectin type III domain-containing tick gut protein (Ixofin3D) was shown to interact with *Borrelia* proteins (76). Similarly, a dystroglycan-like protein was identified on the surface of tick gut epithelium (77). RNAi silencing of these proteins demonstrated their essential role in the migration of *Borrelia* through the gut epithelium toward tick salivary glands and ultimate transmission to the host. Likewise, in a *D. variabilis* cell line infected by the intracellular bacterium, *A. marginale*, initial differential transcriptomic studies identified four tick genes involved in cell infection and *Anaplasma* trafficking through the tick. RNAi silencing on the whole infected tick revealed their role in the regulation of infection and transmission (78). Potential applications in anti-tick vaccines have been explored (78). Recently, one protein, subolesin was tested as a potential vaccine candidate (30). This tick protein is particularly interesting because it is well-conserved among tick species and it is an ortholog of akirin, known to function as a transcription factor for NK-kB gene expression and regulation of the innate immune response (79).

To move to the **salivary glands**, where pathogens will be inoculated into the host, pathogens need to pass through the hemolymph and face innate immune defenses. Tick-borne pathogens developed different strategies to escape the tick immune system and allow their transmission to the vertebrate host. Some have been particularly well-explored like *Borrelia* and *Anaplasma*, others like *Rickettsia* and *Babesia* deserve further investigation (43). The Imd pathway was activated upon interaction with *B. burgdorferi* and *A. phagocytophilum*, both transmitted by the *I. ricinus* complex, and it limits their proliferation (34). However, there are differences in the activation of this pathway between insects and ticks (34).

Transcriptomics and proteomics studies of uninfected and infected ticks revealed up and down regulation of molecules upon infective blood meal (17, 80, 81). Some specific tick molecules are used by the pathogens to facilitate their development and persistence within the tick. This topic has been particularly well-studied in *Borrelia*-infected ticks. First, within the tick gut, a bacterial protein, OspA, interacts specifically with a receptor, TROSPA (tick receptor for Outer surface protein A) (82). In addition, the presence of *Borrelia* upregulates some specific tick proteins to facilitate their survival within the salivary glands. A salivary gland protein, Salp25D, is a gluthathione peroxidase that helps *Borrelia* to establish within the gut (83, 84). Another tick protein, tre31 is induced in the gut and interacts with a *Borrelia* lipoprotein BBE31 (85). The use of RNAi demonstrated the essential role of these proteins for the colonization of the tick by *Borrelia*. The most studied tick saliva protein is likely Salp15, which was shown to interact with OspC and facilitate the transmission of *Borrelia* to the vertebrate host (86). This protein targets different immune cells of the vertebrate host: dendritic cells, T cells, keratinocytes, B cells (87). Another model particularly well-investigated is *I. scapularis* infected with *A. phagocytophilum*. As an intracellular bacterium, the interaction tick-bacteria has been first analyzed in the tick hemocyte cell line ISE6 (88). Then, a combination of transcriptomics and proteomics on nymphs and adult female midguts and salivary glands revealed a major impact of *Anaplasma* on the apoptosis process. The bacteria inhibit this pathway to facilitate their survival within the tick (89). In one hand, they increase histone modifying enzymes (90) and on the other hand inhibit gluconeogenesis and activate glycolysis (91). Similar “omics” approach has been undertaken to analyze the development of *Babesia* (19) in ticks. It also led to the identification of different tick molecules. Recent reviews describe the major role of tick saliva in the virulence and transmission of TBPs to the vertebrate host (92–94).

### Duration of Tick Feeding to Pathogen Transmission

As examined for *I. ricinus*, tick-borne pathogen enzootic cycles are maintained through complex interactions of multiple factors that include abundance and diversity of hosts, larval tick density, likelihood of tick encounters with preferred hosts, pathogen effects on host and tick behavior, aggregation of ticks among hosts, pathogen transmission efficacy, success of larvae molting to nymphs, and success of nymph host seeking, feeding and pathogen transmission (95, 96). Pathogen transmission depends on the tick establishing successful blood feeding and avoiding host defenses of pain, itch induced grooming, hemostasis, and immune rejection at the host cutaneous interface (13, 97, 98).

Ixodid adult female tick blood feeding is divided into the initial slow phase of a week or more, during which weight increases ten-fold, followed by a rapid engorgement period of ~12 to 24 h, during which the tick increases to 100 times or more the unfed weight (53). Cellular and molecular developmental and physiological interactions occur between the pathogen and the tick vector, including during the blood feeding phase (41, 99). Impacted by developmental events within the tick, an important parameter related to these phenomena is the duration of tick blood feeding prior to successful passage of an infectious agent into the bite site and establishment of infection (9, 100). These parameters have practical implications for disease prevention. Reducing risk of tick-borne infections is predominantly an individual responsibility and relies significantly on use of repellents, protective clothing, and checking one's body for ticks (101, 102). Therefore, knowing how long a tick must feed prior to transmission of specific pathogens can help inform potential effectiveness of specific prevention measures, such as tick checks.

Argasid tick blood meals are much smaller than those of ixodid ticks, and they are completed in approximately 1 to 2 h, depending upon the life cycle stage (52). The argasid, *O. turicata* transmits relapsing fever, *Borrelia turicata*, spirochetes within 15 to 40 s of initiating feeding (103). Rapid transmission and host infection are attributed to preadaptation of *B. turicata* in tick salivary gland to the vertebrate host environment (103).

Variations occur in duration of ixodid tick feeding prior to transmission of a specific tick-borne pathogen as well as for different pathogens (100). *B. burgdorferi* transmission is well-studied in regard to development within the feeding tick and transmission to a vertebrate host by the North American vector, *I. scapularis* (100, 104, 105). Nymphs and adults transmit spirochetes, with nymphs transmitting the majority of infections (106, 107). Determinants of transmission include a six-fold increase in the number of spirochetes in tick gut from initiation to 48 h of blood feeding followed by a rise at 72 h of engorgement of salivary gland spirochetes by 21-fold (105). Using larval xenodiagnoses, spirochete transmission from infected nymphs occurred in one of 14 hamsters at 24 h of feeding, 5 of 14 at 48 h, and 13 of 14 after 72 h or longer of engorgement (104). However, due to different *Ixodes* vector and different pathogen species in Europe, the transmission delay can be shortened, especially for *B. afzelii* (108). The pathogen can already be transmitted after 24 h.

*Anaplasma phagocytophilum* was transmitted to 9% of experimentally infested mice by 24 h, 76% by 36 h, and 85% at 50 h of tick feeding (104). A study that examined two time points found *A. phagocytophilum* transmission did not occur by 40 h of tick attachment; however, 100% of mice were infected by 48 h (109). *Babesia microti* sporozoite transmission occurred in 9% of hamsters at 36 h and 50% after 54 h of infestation (110). A *B. burgdorferi* co-transmission study found that 71% of hosts were positive for *B. microti* infection at 54 h of tick attachment (104). *A. phagocytophilum* and *B. microti* can occur individually as co-infections with *Borrelia burgdorferi*, resulting from the bite of a tick infected with both microbes (111, 112).

*B. burgdorferi, A. phagocytophilum* and *B. microti* are transmitted by ticks of the nearly globally distributed *I. ricinus* species complex that includes *I. ricinus, I. persulcatus, I. scapularis, I. pacificus* and additional species (113). These tick species are also competent vectors for tick-borne encephalitis (TBE) virus (114). TBE virus exists as three geographically defined pathogenic subtypes in endemic foci from Japan across Eurasia to France (115, 116). Powassan virus is the reemerging North American representative of the TBE virus groups, and it occurs as two lineages (117, 118). Powassan virus was also isolated in Russia (119). *I. scapularis* nymphs transmitted Powassan virus to mice by 15 min of initiating feeding with maximum transmission efficiency at 180 min (120).

*Borrelia miyamotoi* is a relapsing fever spirochete also transmitted by members of the *I. ricinus* species complex and occurs over the same geographic regions (121, 122). Transmission risk increased with infestation by a single infected *I. scapularis* nymph from 10% after 24 h to 73% at 72 h (123). A single *I. scapularis* infected nymph transmitted *B. mayonii* with a 31% probability of infection at 72 h of engorgement with no evidence of transmission at 24 or 48 h (124).

Unclear is the process by which reactivation of spotted fever group rickettsial virulence occurs within the vector tick during the period from attachment to the host through blood feeding (125). Reactivation occurs during the 6 to 10 h infected ticks feed before rickettsiae are transmitted (126). In addition to blood feeding, virulence can also be restored by exposing unfed, infected ticks to 37°C for 24 to 72 h (127). Overall, bacteria and parasites need to migrate and undergo development within the ticks explaining a delay in pathogen transmission to the vertebrate host, while viruses are transmitted as soon as the tick blood meal is initiated (8, 9).

### Salivary Glands: A Key Organ in Pathogen Transmission

The structure of Ixodid tick salivary glands is composed of three types of acini in females and four types in males (128–131). Type I acini occur in all ixodid life cycle stages; these acini lack secretory granules; and, they contribute to maintaining off host water balance by production of hygroscopic saliva (130). Type II and III acini both increase in size and granularity over the course of engorgement combined with release of granular contents (129, 130).

Number and diversity of salivary gland derived proteins were greatly increased by application of reverse genetics strategies that included sequencing of full length cDNA libraries in combination with increasingly powerful bioinformatic and proteomic analyses tools (17, 132). Next generation sequencing platforms combined with proteomics informed by transcriptomics revealed even greater salivary gland gene product complexity (133–135). Transcribed salivary gland gene analyses revealed differences between and within prostriate and metastriate species; gene transcription changes during infection with tick-borne pathogens; widely conserved multigenic families; pluripotency and redundancies in gene products that target specific host defenses; and, saliva composition changes occurring during the course of feeding, including members within a gene family (135–142). Analyses can now be performed on a single pair of salivary glands rather than on pooled glands, revealing individual tick specific properties and variations within a population (17, 143). Host species related specific salivary gland gene expression adaptations also occur (144). While these gene expression studies focused on proteins, salivary glands also produce non-protein compounds, purine nucleoside and prostaglandins, with important biological activities (145). More recently, non-proteinaceous molecules like **small RNAs** (miRNAs and small-interfering RNAs) were described as gene regulators. They are produced after cleavage by the DICER protein and they bind to complementary mRNA target leading to gene silencing. They have been studied in several tick species. MiRNAs can be involved in the regulation of tick development (146) or blood feeding (147). MiRNAs have been detected in *I. ricinus* saliva and might be excreted in exosomes that could modulate the vertebrate host homeostasis at the skin interface (148). Interestingly, some of these miRNAs have been characterized in *I. scapularis* salivary glands during the transmission of Powassan virus to mouse model (149). They have been also detected in tick hemocyte ISE6 cell line infected by the bacterium, *A. phagocytophilum*. A specific miRNA, isc-mir-79, was particularly upregulated. It targets a transmembrane protein belonging to the Robo immunoglobulin family involved in inflammatory processes (150).

Transcriptomic and proteomic analyses also identified a multitude of salivary gland protein predicted biological activities, molecular targets, and functions that include modulators of host pain and itch, vasodilation, platelet aggregation, coagulation pathways, innate and adaptive immune effectors and regulators, and wound healing (92, 135, 151–153). Attention is increasingly focused on characterizing major groups of broadly bioactive molecules present in saliva cross multiple tick species, such as cystatins and Kunitz inhibitors (154). Histamine-binding lipocalins (155) and releasing factor (155) are examples of targeted differential effects of tick saliva on a host response mediator during different phases of blood feeding. Although the number of identified salivary gland genes and miRNAs continues to increase, the fundamental problem remains of linking individual molecules to specific biological activities at the tick-host-pathogen interface.

Differential production of bioactive molecules correlates with anatomical and histological changes occurring in tick salivary glands during the course of blood feeding; however, regulatory events controlling saliva production require continued study.

## Vertebrate Host

Immune tolerance to tick-borne pathogens differs whether the vertebrate is a reservoir (a host, source of infection to tick and not clinically ill) or a susceptible host (a host, that ticks feed on in nature) (156). This susceptible host can be either clinically ill or neutralizes the pathogens and is only serologically positive (157).

In this process of tolerance to the intruder (the tick and the potential pathogen), the skin plays a key role by its immunity (158) and its microbiome (159). In addition to its role as an inoculation site, it has been shown to be a site of persistence in some insect-borne diseases such as malaria parasite (14) and for trypanosomiasis (16, 160, 161). In TBDs, Lyme borreliosis has been the most investigated for this aspect (13). Additional studies on other TBDs deserve further investigations to conclude to a common role of the host skin in pathogen persistence.

### Structure and Immunity of the Skin

The skin is the largest organ and more than just a physical barrier. It is structured into three major layers: the epidermis, the dermis, and the hypodermis (162). The **epidermis** is the outermost layer with a stratified epithelium, mainly constituted by tissue resident cells, the keratinocytes, which undergo sequential differentiation, and melanocytes. Keratinocytes are integral components of the skin innate immune system (158). They have been studied for their role in secretion of the defensins (163–165), cathelicidin (166, 167), and control of skin infection. It is well-documented that these antimicrobial peptides (AMPs) increase adaptive immune responses (165, 168). Langerhans cells reside mainly in the epidermis and represent 2–8% of the epidermal cell population (169). The **dermis**, which underlies the epidermis, is a connective tissue with fibroblasts as resident cells secreting extracellular matrix, collagen and proteoglycans (170), giving the dermis its toughness and resilience (158). Dermis is well-drained by both blood and lymphatic vessels, which facilitate circulation of immune cells. It is therefore rich in migrating immune cell populations: dendritic cells, mast cells, macrophages, T lymphocyte subsets (CD4 T cells and CD8 T cells), natural killer cells and innate lymphoid cells (ILCs) (171). All these cells possess a strong ability to recognize pathogens and to be activated (158, 172). Below these two layers, **adipose tissue** constituted of subcutis, **hypodermis** and dermal white adipose tissue (dWAT). The dWAT within the reticular dermis, is involved in thermoregulation, hair cycling, wound healing and most recently in immunity (173). Its main cells are adipocytes, secreting adipokines and AMPs, but also immune cells. Adipocytes display various pattern recognition receptors and then produce various cytokines and chemokines (173, 174). The dWAT also surrounds the hair follicle. A specific interplay exists between the hair follicle cycle and the intradermal adipocyte. PDGF (platelet-derived growth factor) secreted by immature adipocytes, activates the growth of the hair follicle (175). Recently, the hair follicle has been shown to harbor a complex microbial community due to its moist and less acidic environment compared to the epidermal surface. This community is regulated by specific AMPs and constitutes an immune-privileged site, potentially used by persistent pathogens (176) (see below).

Our improved knowledge of the structure and immunological function of the skin provides the framework for understanding tick and tick-borne pathogen induced immune tolerance. To protect from invaders the skin has developed a complex network of cellular interactions that ensure host defense and preserve homeostasis (158, 177). This network relies on (1) innate immunity with the resident skin cells of the epidermis and the dermis, and more specific immune cells like Langerhans cells, mast cells, dendritic cells, macrophages and innate lymphoid cells (ILCs), and (2) adaptive immunity which relies on various subpopulations of T cells (169). Within this structure different appendages like hair follicles, sebaceous glands and sweat glands participate in skin homeostasis and protection.

The role of macrophages, and more particularly of neutrophils, has been investigated in vector-borne diseases (178, 179). While macrophages and neutrophils are studied in the contexts of infectious diseases and tissue repair, the roles of lymphocytes are reevaluated at the skin interface. ILCs respond to epithelium-derived signals (cytokines, cell-surface receptors and lipid mediators) and therefore constitute an important actor of skin homeostasis. They divide into three subgroups according their cytokines profiles. The secreted cytokines modulate the immune response and ILC functions overlap and complement T cells (11, 180, 181). Then, acquired immunity relies on antigen-specific T cells. First, effectors T cells are generated upon acute infection leading to a long lasting immunity in the skin, with the development of resident-memory T cells (T_RM_) (182). In adult human skin, memory T cells are four times more important than in peripheral blood and four distinct populations of these T cells have been identified according to their surface receptors (183). These skin-homing T cells are produced in skin-draining lymph nodes, where they acquire specific chemokine receptors (CCR4, CCR8, and CCR10) and leukocyte integrins to come back to skin tissue (172). Regulatory T cells play a key role in homeostasis and inflammation in the skin, where they are particularly abundant. These cells are also part of the resident cell population and interact with fibroblasts and Langerhans cells (10). How this complex immune network control so efficiently tick-borne pathogens at the skin interface needs to be elucidated.

### Tick Induction of Cutaneous Immune Tolerance

When ticks introduce their mouthparts into the skin tissues, they lacerate the epidermis and the dermis due to their telmophage bite that induces a blood pool in the dermis, where they inject saliva and consume blood. While the presence of chitin on mouthparts should trigger an immune response (184), it seems that the tick succeeds once again in escaping the host immune system. Tick saliva is responsible of this immune escape (185, 186). Indeed, it has been known for years that tick saliva exerts a potent local immunosuppression by secreting a large array of molecules that target multiple elements of the immune system (93, 153, 187, 188).

Salivary gland transcriptomes and proteomes have shown how tick saliva modulates vertebrate host innate and adaptive immune responses and wound healing (134, 189). Increased examination of mediators, cells, and crosstalk among these elements will greatly enhance or understanding of events occurring at the tick-host-pathogen interface. In addition to the receptor populations, cells, cytokines, chemokines, and interleukins that are well-studied in the context of tick induced modulation, emphasis can be placed upon less well-studied cells in the tick-host relationship, such as keratinocytes, melanocytes, fibroblasts, adipocytes, and innate lymphoid cells and mediators such as alarmins. Resident skin cells (keratinocytes, fibroblasts, and adipocytes) deserve further investigation based on the increasingly recognized roles of these cells in immunity that are emerging (15, 190, 191). Innate lymphoid two cells are also of interest relative to potential cytokine polarization to a Th2 profile at the tick bite site. These innate immune cells have not been studied so far in the context of TBDs. Due to their role in the regulation of the innate immune response at the skin interface (180, 181, 192), they must also play a role during tick feeding and inoculation of pathogens. It may be particularly relevant to analyze these ILC2 cells, since it is well-documented that Th2 lymphocyte response is induced during the introduction into the host of tick-borne pathogens such as *B. burgdorferi* (193, 194) and spotted fever group rickettsiae (195).

Finally, since tick saliva modulates pain and itch responses, the interactions of saliva with dermal peripheral nerve endings deserve investigation since the role of the nervous system and its connection with the immune system is unknown during the tick blood feeding that lasts for days (196).

### Tick Attachment and Feeding Site: Role of Tick Saliva

Understanding tick-host-pathogen interactions requires characterizing and defining the biological activities of tick saliva molecules during the course of feeding and infectious agent transmission. Ixodid tick feeding presents unique challenges due to larvae and nymphs blood feeding for days while adult feeding may require more than a week (53). Host defense systems evolved to reduce or eliminate insults on homeostasis; however, ticks developed effective countermeasures to host pain and itch responses, hemostasis, innate and adaptive immunity, and wound healing (92–94, 97, 98, 135, 154).

Tick-borne infectious agents exploit tick saliva modulation of host defenses that create an immune tolerant bite site environment favorable for pathogen transmission and establishment (13, 92, 153, 193). Balance is not static between host immunity to tick feeding and tick modulation of host immune defenses, as occurs during repeated infestations (97, 98, 154). Acquired resistance to tick bite represents a tipping of that balance toward host immune dominance that results in impaired tick engorgement, blocked molting, and tick death (197–202). While tick modulation of host defenses can facilitate pathogen transmission, acquired resistance to tick bite significantly inhibited *Dermacentor andersoni* transmitted infection with *Francisella tularensis* type A (203). Development or absence of acquired resistance depends upon the tick species and host species infested (197, 200, 204).

The complexity of tick salivary gland derived molecules increased dramatically during the past five decades due to the emergence of transcriptomics, next generation sequencing, and quantitative proteomics (17, 135). Early studies relied on isolation and biochemical characterization of individual bioactive molecules from salivary glands of feeding ticks (132, 205–207). Valuable insights were obtained about saliva activities. Biochemical isolation and characterization combined with analysis of biological activity studies were generally labor intensive; required large amounts of starting material; and, depended upon activity identification assays at each fractionation step.

In addition to secreted saliva molecules into the host skin, attachment cement is a salivary gland secretion that serves as a holdfast structure and sealant of the bite site whose production starts within minutes of host attachment and, depending on the tick species, occurs in different patterns during the course of blood feeding (131). Attachment cement production is linked to distinct cell types within type II and III acini (128) along with a possible contribution from type I acini (130). Tick saliva can be trapped in attachment cement (208) along with tick-borne pathogens (209).

To conclude, tick saliva induces a transient potent immune tolerance at the bite site to avoid its rejection. Pathogens, when present in infected ticks, behave as opportunistic microorganisms at the skin interface and take advantage of this immunosuppression (210).

### Tick and Host Pharmacology

Concerning the pharmacomodulation of tick saliva at the skin interface, successful tick blood feeding depends upon inhibiting host hemostasis and wound healing that allows access to a continuous supply of blood. Ticks evolved salivary secretions that inhibit platelet aggregation and activation, act as vasodilators, and block the action of multiple components of the coagulation cascade (154, 188, 211). Hemostasis is also the first phase of the multi-step process of acute injury cutaneous wound healing (212), a process that ticks regulate primarily during hemostasis and inflammatory response phases (97, 98, 135). Argasid and ixodid ticks evolved multiple, redundant strategies to counteract platelet aggregation and activation of the different vertebrate host species from which individual tick species are capable of obtaining a blood meal by blocking platelet integrins, binding platelet activating molecules, or inhibiting protease activated receptors (154, 188, 211, 213, 214). Tick saliva contains numerous Kunitz domain serine protease inhibitors that disrupt platelet aggregation and coagulation cascade activation (154, 188, 213). Saliva Kunitz inhibitors act upon coagulation cascade factors Xa and thrombin due to their activation of platelets and multiple coagulation pathway enzymes (188, 215–218). Tick saliva contain few vasodilators (214).

Itch-induced grooming is a threat to ticks that are continuously attached and blood feeding for several days. The relationships among tick feeding, host acquired tick resistance, and the itch response were described for *Rhipicephalus (Boophilus) microplus* infestations of cattle either restricted from self-grooming or allowed to groom, lick, freely (219–222). Experimental infestations of cattle restricted from grooming resulted in an average yield of 33% engorged adults whilst the adult recovery from animals allowed to groom freely was 9% (219). Grooming-induced tick mortality was directed primarily toward larvae within the first 24 h of infestation (221) that resulted in larval losses of up to 54% (223).

Infested cattle developed acquired resistance to *R. (Boophilus) microplus* that upon reinfestation resulted in reduced tick feeding weight, yield of adults, and egg mass (220). Acquired resistance was linked to a cutaneous allergic hypersensitivity response characterized by an influx of eosinophils and development of serous exudates that trapped ticks (220). Significantly, highly tick resistant cattle blood histamine levels peaked at 48 h after applying larvae and persisted for 8 days, while little or no changes occurred in blood histamine concentrations for infested, non-resistant cattle (220). Histamine and its receptors are commonly associated with the temporary sensation of cutaneous itch (224, 225), and anti-histamines are well-recognized treatments for itch (226). Serotonin, 5-hydroxytryptamine, elicits both pain an itch responses independent of histamine with only an itch response stimulated at lower concentrations (227, 228). Recent reviews focus on molecular and neural mechanisms of itch that include peripheral initiation of the response, sensory neurons, mediators, receptor, central nervous system perception, scratching response to eliminate an acute stimulus, and shared features of itch and pain responses (224, 225, 229, 230).

Cutaneous injury, such as a tick bite, results in mast cell release of histamine, serotonin, cytokines, chemokines, and proteases that mediate vasodilation, inflammatory cell influx, and stimulation of itch receptors (225, 231). Platelet aggregation in response to injury also releases histamine and serotonin (232, 233).

Regulating the actions of histamine and serotonin are central to tick modulation of the itch response. Chinery and Ayitey-Smith (234) reported that *Rhipicephalus sanguineus* salivary gland extract contains a histamine blocker. Three histamine binding proteins were found in *R. appendiculatus* saliva and each had one high and one low affinity binding site for histamine (235, 236). Similar dual receptor binding affinity active sites occur on a histamine binding protein in *D. reticulatus* salivary glands that was demonstrated to have one high affinity histamine receptor and a low histamine affinity receptor that bound serotonin with high affinity (155). These two different receptors on one saliva protein bind two important mediators of acute itch to tick bite.

Histamine and serotonin directly impact tick feeding. Upon exposure to histamine and serotonin in a blood meal, *D. andersoni* female salivation and blood uptake were inhibited (237). Mechanisms mediating acquired tick resistance remain to be fully defined, elevated bite site histamine levels negatively impact tick feeding and induce host grooming (238, 239). Elevated histamine can also be linked to the basophil rich inflammatory cell influx at tick attachment sites and development of epidermal hyperplasia that disrupts tick feeding (240–242).

### Basophils and Acquired Resistance to Ticks

Basophil responses at tick attachment sites are linked to the phenomena of acquired host resistance to tick infestation and the immunological basis underpinning the response. Two studies are foundational in linking host immune responses to tick bite. Jellison and Kohls (243) hypothesized that host immunity was responsible for poor tick feeding on rabbits repeatedly infested with adult *D. andersoni* and for development of crust-like lesions at tick attachment sites. In a foundational study, Trager (244) observed that guinea pigs developed resistance to infestation with *D. variabilis* larvae after one infestation, and that resistance was expressed during a second infestation as reduced tick engorgement, death of ticks, discolored feeding ticks, and small blisters at attachment sites. Histology of first exposure larval attachment sites was characterized by slight epidermal thickening with little cellular reaction, while second exposure bite sites contained large numbers of polymorphonuclear leukocytes, few eosinophils, and epidermal thickening extending below the inflammatory cell containing “mass” (244).

Allen (240) established that the cellular response in vesicles in hyperplastic epidermis beneath larval mouthparts on guinea pigs expressing acquired resistance to *D. andersoni* consisted of high concentrations of basophils attributed to a cutaneous basophil hypersensitivity response. During a repeated infestation in which acquired resistance was strongly expressed, histologic changes at attachment sites consisted of epidermal acanthosis and acantholysis; dermal influx of eosinophils, lymphocytes, and macrophages with no increase in mast cells; and, numerous basophils accumulating in vesicles beneath mouthparts (245). Likewise, cattle expressing acquired resistance to *I. holocyclus* developed basophil rich inflammatory responses at tick attachment sites (245). Basophils also accumulated at tick bite sites in humans (246).

In an elegant and definitive study that maintained the functional integrity of mast cells, selective ablation of basophils established their non-redundant role in murine acquired resistance to infestation with *Haemaphysalis longicornis* larvae (247). Mast cell deficient mice developed resistance to *D. variabilis* larvae after repeated infestations; however, their mast cell sufficient counterparts developed a more marked resistance, suggesting a minor role for mast cells in this tick-host association (248). Basophils were detected by ultrastructural examination of *D. variabilis* larval attachment sites on mast cell deficient mice after three infestations (249). In contrast to *D. variabilis* larval infestations, mast cell deficient mice of the same strain failed to develop acquired resistance to *H. longicornis* larvae (250). Tabakawa et al. (251) subsequently established that histamine derived from basophils infiltrating the tick bite site, not mast cells, were responsible for expression of acquired resistance. Recruitment of basophils to the tick attachment site was linked to interleukin-3 produced by skin resident memory CD4+ T lymphocytes (252). The central role of basophils in acquired resistance to ticks was recently reviewed (241, 242).

Does acquired resistance to tick infestation alter pathogen transmission? Rabbits expressed acquired resistance after one infestation with uninfected *D. variabilis* adults that provided significant protection against transmission of the bacteria, *Francisella tularensis* type A, by an infestation with infected *D. variabilis* nymphs (253). The mechanism by which resistance to this highly virulent pathogen was expressed remains to be determined. One possibility is that the inflammatory reaction at the bite site creates a milieu that reduces infectivity or kills the bacteria.

Basophils and mast cells were recently reviewed in the regard to similarities and differences in their biology, roles in host defense and disease pathogenesis, and availability of specific molecular tools to distinguish the effector functions of these two important cell types (254–258). Roles of mast cells and basophils in cutaneous immunity and inflammation were reviewed in the context of Th2 responses, innate lymphoid cells, and eosinophils (259, 260). Basophil function as antigen presenting cells for Th2 responses remains a topic of ongoing study (242, 260).

### Skin Microbiome

The skin microbiome is part of skin immunity (261). Its major role increased lately in studies on skin inflammation (166, 262). The vertebrate host and its microbiota are now considered as a holobiont or a hologenome (263, 264). The cutaneous surface, the largest organ of 1.8 m^2^, is colonized by a diverse population of microbes, ranging from bacteria, mites, yeasts, and viruses (3). The composition of the human skin microbiota varies according to moist, dry or sebaceous microenvironments. These symbiotic microorganisms occupy the skin surface and specific niches, such as hair follicles and sebaceous glands. A precise 3D mapping by mass spectrometry and 16S rRNA sequencing revealed the impact of the skin surface environment on the composition and chemistry of human skin microbiome (265).

Various forms of interaction exist between these microorganisms, encompassing mutualism, parasitism and commensalism depending on the context (159). In addition, these microorganisms cooperate with the host immune system to maintain skin homeostasis. Immune tolerance to these commensal microbes is essential. It seems to take place in neonatal life with help of Treg cells as shown in a mouse model colonized specifically with a *Staphylococcus epidermidis* transformed to express a model 2W peptide coupled to a fluorescent protein (266). How skin Treg cells induce tolerance to commensal antigens remain to be investigated, but it seems to be different from the mechanisms operating in the intestine and then to be tissue specific. Scharschmidt et al. speculate that the hair follicles could be the site where Treg reside since both, the hair follicle morphogenesis and Treg production take place at the same time. A hair-follicle related chemokine would attract the Treg into these appendages (266). The skin microbiome also educates the innate immunity by shaping the expression of IL-1 alpha, complement system and AMPs (mainly cathelicidin and beta-defensin). Therefore, commensal microbiota is considered as an adjuvant to the immune system (267). For example, *S. epidermidis*, a major constituent of the skin bacteria, participates in innate immunity by secreting its own antimicrobial peptides to control pathogens at the skin interface, also creating a favorable environment for itself (261).

The skin microbiome is an interesting and important area for future investigation in the context of VBDs. While several studies have been performed on the interaction of mosquitoes and skin microbiome, they are mainly focused on the role of commensal bacteria on mosquito attractiveness (268–270). Early studies revealed the role of *Brevibacterium epidermidis* of human host on the attractiveness of *Anopheles*, the vector of malaria parasites (271, 272). Very few studies have investigated the role of skin microbiota on pathogen transmission at the skin interface. They concern *Leishmania* parasites (273, 274). Germ-free mice develop larger lesions, a higher parasite load and their macrophages are less efficient to kill intracellular parasites (274). It has also been shown that germ free mice have an impaired immune response against *Leishmania* parasites that can be partially rescued by inoculation of the commensal skin bacteria, *S. epidermidis* (273). *Leishmania* might induce dysbiosis; this rupture in skin homeostasis would lead to the recruitment of neutrophils and IL-1 beta secretion increasing the severity of the disease (275). Surprisingly, up to now no such studies have been performed on ticks and tick-borne pathogens to analyze the role of host microbiota in tick-attractiveness and pathogen transmission. This last aspect is particularly relevant in TBDs, since the tick lacerates the host skin, creates a feeding pool and remains for several days attached to the host skin (276). It is very likely that the microbiota penetrates from the surface of the skin, deeper in the dermis and might contribute to local immunomodulation during the bite and pathogen transmission. The role of skin microbiota is definitely a research area that deserves further investigation.

## Tick-Borne Pathogens

### A Worldwide Increase

Due to major climate change, modifications of the ecosystem and global trade, tick population and TBPs increase worldwide (277–280). Ticks transmit a diverse array of pathogens; therefore, the public health impact of established, resurging, and emerging tick-borne infectious agents is increasing (98). Changing geographic ranges of tick species is associated with movement of tick-borne infections as well with the potential for creation of new endemic areas for diseases (281–283). Some of TBP are considered as emerging pathogens however this increase is also likely due to better detection methods, awareness of health practitioners and patients and closer contacts between ticks and populations.

The high incidence of Lyme borreliosis in northern hemisphere (107) has likely hidden other TBDs in humans with lower incidence like anaplasmosis, relapsing fever associated with *B. miyamotoi* among others. Since the 1990s, molecular tools allowed the identification of a number of microorganisms such as *Neoerhlichia mikurensis, B. miyamotoi* and different *Rickettsia* species within ticks (157). Facing clinical pictures different from Lyme borreliosis in patients, biologists looked for these pathogens by direct diagnosis in blood and tissues by PCR. Consequently, the panel of TBDs in human significantly increased, especially in patients suffering from immunosuppression (284–287). The immune status of the patient is a key element in the outcome of disease. Due to concurrent medical procedures and conditions (e.g., cancer and grafts), the number of immune-compromised patients has increased. It explains why new TBPs are detected in these patients, improving indirectly the knowledge on tick-borne diseases and potential pathogen isolation as shown for *B. miyamotoi* (288, 289) or *N. mikurensis* (290). In parallel, serological surveys performed in tick endemic areas revealed that the number of exposed people to TBP is significantly higher, as shown by the seroprevalence against TBPs, increasing worldwide (157).

### Molecular and Cellular Tools to Identify and Study Tick-Borne Pathogens

Molecular tools such as next generation sequencing and functional “omics” (genomics, transcriptomics, and proteomics) for identification of potential emerging tick-borne pathogens are essential to make a direct detection of pathogens in tick or in the vertebrate host (17). PCR can be developed rapidly and can be tested on different matrices (whole tick, blood, skin biopsy or biological fluids). Application of molecular techniques was the basis for the reversed discovery of tick associated microbes that were subsequently recognized as human pathogens: *B. miyamotoi* (291), *N. mikurensis* (292), *Rickettsia helvetica* (293), *R. monacensis* (294), and other *Rickettsia* species identified by genomic methods (295). High throughput sequencing of the microbiomes of *I. scapularis, D. variabilis*, and *A. americanum* from a single site in New York State resulted in identification of nine new viruses (296). This study design was expanded to multiple sites in Connecticut, New York, and Virginia with the detection of nine previously characterized viruses and 24 presumably novel viral species (297). New microbial species were detected in Western Europe when the *I. ricinus* microbiome was analyzed by next generation sequencing (298).

Single cell technologies, for example, flow cytometry to analyze cell surface markers or single-cell RNA sequencing (sc RNAseq) should also greatly help to better understand host-pathogen interactions and identify key molecules involved in cell-cell interaction. The complexity of the immune system network involves in these interactions requires complementary techniques and comprehensive analysis (18). Two photon intravital imaging visualized the interaction of different parasites in the skin: the persistence of *Plasmodium* parasites in the hair follicle (299), the role of neutrophils in *Leishmania* (179) and *Trypanosoma brucei* infections (178). Very few studies have been performed with TBDs (300). For example, laser microdissection coupled to scRNA seq (18) could be used in infected and control skin to localize the site of pathogen persistence and better appreciate the respective role of dermal adipocytes and hair follicle environment.

### Tick-Borne Pathogen Immune Modulation of Vertebrate Host: Inoculation, Multiplication and Persistence

Tick-borne pathogen modulates host defenses in a manner very similar to, or complementary to, tick induced host immune modulation. The immune modulation is involved in different events of the vertebrate host infection: pathogen transmission and pathogen persistence.

Two tick-pathogen systems have been particularly explored: *Rickettsia*-*Dermacentor* et *Ixodes*-*Borrelia*. The **spotted fever group rickettsiae control host defenses, and its competent vector**, ***Dermacentor andersoni***, also controls host immune defenses in a very similar manner to that of the rickettsiae. Basically, the tick vector and the pathogen complement each other in manipulating host defenses (125). Transmission, pathogenesis and evasion of host defenses by spotted fever group rickettsiae reviewed by Sahni et al. (125) noted that knowledge was incomplete relative to the influences exerted by the tick vector in transmission and establishment of infection by rickettsiae. *D. andersoni*, Rocky Mountain wood tick, is a competent vector of *R. rickettsii* (301). Ability of this tick to modulate host innate and adaptive immune defenses has been the subject of multiple studies (302–305). Immunomodulatory molecules contained in tick saliva are introduced into the host prior to transmission of rickettsiae, creating a cutaneous environment that is favorable for both blood feeding and pathogen transmission (305). The tick vector is attached to the host for 6 to 10 h prior to transmission of *R. rickettsii* (100).

Here, we examine potential synergies between host immune evasion induced by rickettsiae and that induced by *D. andersoni* feeding. Immune elements controlling rickettsial infection include: endothelial cells, macrophages, dendritic cells, NK cells, innate immune signaling pathways, proinflammatory cytokines, chemokines, CD4+ T lymphocytes, CD8+ T lymphocytes, and antibodies (125). Macrophages and dendritic cells at tick feeding site are initial targets of infection with rickettsiae, and TNF-α and IFN-γ activated macrophages are effectors capable of clearing rickettsiae within these cells (125). Furthermore, endothelial cells activated by TNF-α and IFN-γ are induced to kill intracellular rickettsiae, and CD4+ T lymphocyte derived IFN-γ important in host protection against rickettsiae (125). These host defenses against rickettsiae are modulated by the tick vector. *D. andersoni* salivary gland extracts prepared throughout the course of engorgement suppressed macrophage production of TNF-α and IL-1 and T lymphocyte elaboration of IFN-γ and IL-2 (303).

Severity of disease correlated with whether rickettsiae can survive and proliferate in macrophage-like cells. During rickettsial infection, protective response consists of IL-1, IL-6, and IFN-γ accompanied by inflammatory infiltrates of neutrophils and macrophages (125). In addition to salivary gland extract suppression of macrophage IL-1 and T lymphocyte production of IFN-γ (303), *D. andersoni* infestation suppresses T lymphocyte expression of the integrins LFA-1 and VLA-4, important molecules in leukocyte adhesion to endothelium and movement to sites of inflammation (306) and significantly down regulated vascular endothelial cell expression of ICAM-1 (304). *D. andersoni* saliva proteome analysis identified cystatins and serpins that are putative inhibitors of inflammation (134).

*D. andersoni* nymph infestation induced host cutaneous responses were characterized using a combination of genome arrays and histopathology (134). During an initial nymphal infestation, there occurred a decrease in the number of up regulated host cutaneous genes between 12 and 48 h' post-infestation. This early primary infestation inhibition of transcription and RNA processing was consistent with an observed inhibition of inflammation. Histologic examination revealed that the number of inflammatory cells infiltrating the tick bite site did not increase from 12 to 48 h of a primary infestation. These changes were consistent with inhibition of inflammation during the period when rickettsiae would be transmitted from tick and infection established (134).

A second very well-analyzed tick-host-pathogen relationship is ***I. scapularis* and** ***I*.** ***ricinus* modulation of host defenses in the context of** ***B. burgdorferi***sl transmission and infection. One *Ixodes* protein, Salp15 (Saliva protein 15 kDa) has been particularly explored since it targets different aspects of the host immunity. First, this tick protein is specifically upregulated within the *Ixodes* tick (307) where it binds to OspC (Outer surface protein C), a surface lipoprotein involved in the early transmission of *Borrelia* (308). Then, salp15 targets the different arms of the immune system: innate immunity (complement system and TLR2 receptor) (191, 309) and cellular immunity (the dendritic cells and the T cells) (310, 311).

Once the TBP has been inoculated, two scenarios occur. The vertebrate host possesses a potent immune system and neutralize the pathogens and antibodies are generated in absence of clinical manifestations. This explains the seroprevalence of people or animals regularly exposed to infected tick bites and who do not develop clinical manifestations (312, 313). In some vertebrate hosts, clinical manifestations appear few days or few weeks after the tick bite (107). Interestingly, in the mouse model of Lyme borreliosis, a peak of *Borrelia* multiplication appears 7 to 10 days after the inoculation of bacteria whatever the *Borrelia* species (12). It would be interesting to investigate the potential signification of this peak, perhaps related to generation of immune tolerance in the host skin. In animal models, induction of immune tolerance to pathogens seems to develop and pathogens persist in the skin in absence of antibiotics (314). This has been particularly well-documented for *Borrelia* in the mouse model. The pathogen is alive since it can be reactivated by application of topical corticosteroid that induces a local immunosuppression and local multiplication of *Borrelia* (314, 315). In experimental inoculation of luciferase positive-*Borrelia*, the bacteria can be visualized moving extracellularly in mouse skin for several months (M. Wooten—personal communication).

Since adipocytes have been described as a haven for *Plasmodium* (malaria), *Trypanosoma cruzi* (Chagas disease) and *T. brucei* (sleeping sickness) (15), it would be interesting to investigate whether TBP escape the immune control of the vertebrate host in adipose tissue. More interestingly, the hair follicle, which interacts with the adipose tissue, would be an immune privileged site to explore. It has been clearly shown that the hair follicle constitutes a site of persistence for *Plasmodium* (14). Induced immune tolerance is a result of both tick and pathogen manipulation of the host environment and results in successful establishment of infection. This would be another interesting topic to stimulate future research.

## Conclusions

**Figure 2:**
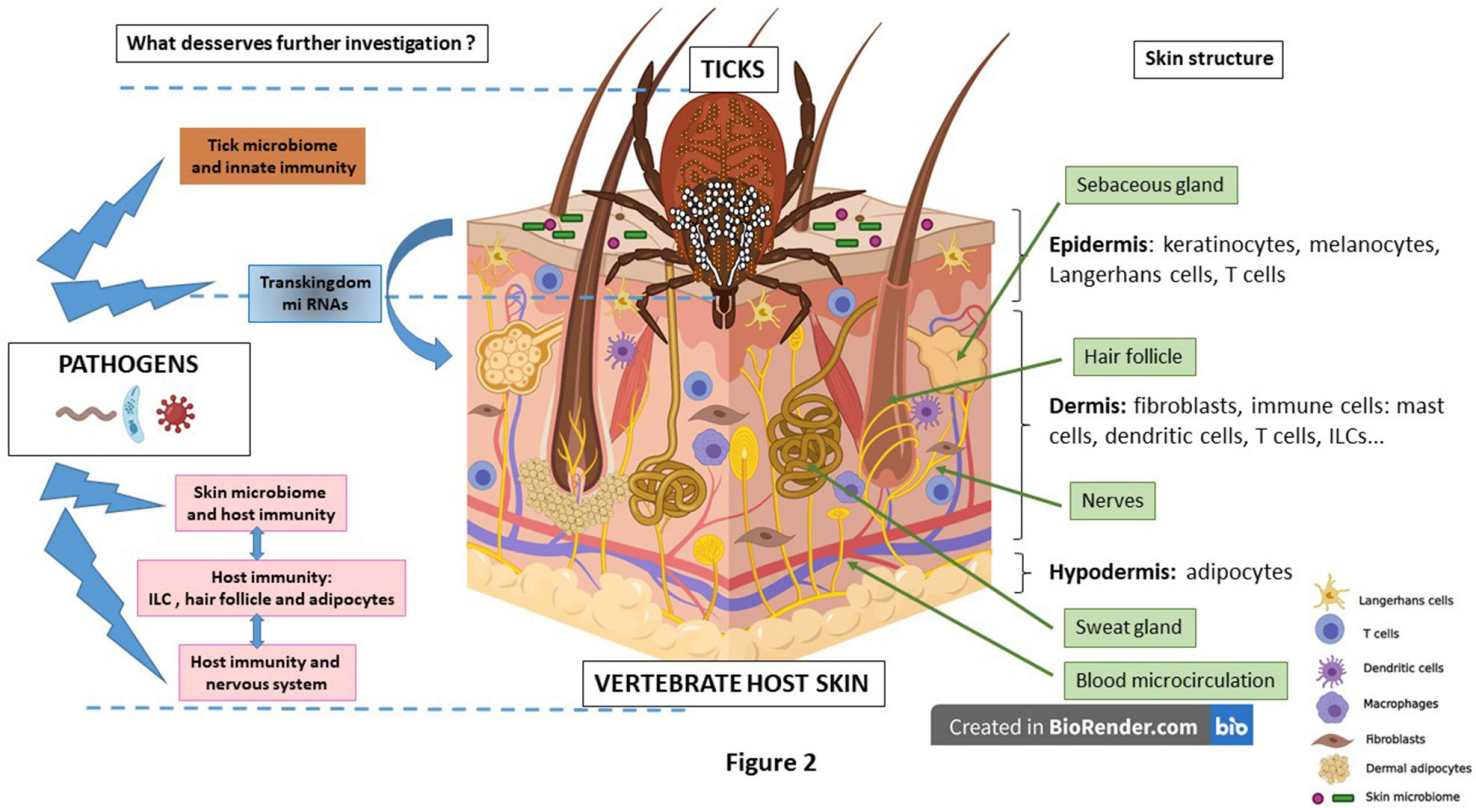
The skin is structured into three interconnected layers. The epidermis, the uppermost layer, mainly composed of keratinocytes accompanied by immune cells such as Langerhans cells and T cells. On its surface, the skin microbiome (bacteria, yeasts and virus) plays a key role in skin homeostasis. The dermis is responsible for resistance and elasticity of the skin. Dermal resident cells are fibroblasts secreting extracellular matrix with numerous immune cells such as dendritic cells, T cells, mast cells, Innate Lymphoid cells (ILCs) and others. Different appendages are present: hair follicles surrounded by dermal adipocytes, sweat glands, microcirculation blood vessels, nerves and sebaceous glands. Then beneath the dermis is the hypodermis that contains numerous adipocytes. During the process of an ixodid, hard tick bite, a feeding pool develops around the tick mouthparts. Saliva is introduced into the bite site and modulates the local host immune response with the goal of avoiding tick rejection. The tick microbiome can be secreted into the skin as exosomes. Likely, transkingdom miRNAs participate in the regulation of the infection. The role of skin immunity and skin and tick microbiomes deserves to be better investigated during the process of pathogen inoculation, multiplication and persistence. Some immune privileged sites like the hair follicle and the adipocytes might help pathogens to better survive within the skin. Recently, the potential role of nerves in the skin immune regulation has been evoked. Created with BioRender.com.

Vector-borne diseases have evolved toward a very complex, multifaceted network (Figure 2) (316). Initially described as a simple triad, vector-pathogen-vertebrate host, additional factors appear to regulate the network like the microbiome (2, 3) and non-coding RNAs (4, 317).

**The vector** by its innate immune system regulates the pathogen population to allow vector survival (19, 73). It also differentiates its microbiome from tick-borne pathogens (2). How this operates is still not clear. **The vertebrate host** can tolerate the tick-borne pathogen and becomes a reservoir (156). This specific relationship between the host and the pathogen is essential for enzoonotic cycle maintenance. Accidental host is more or less susceptible to the pathogen. He can be an immune tolerant host who develops an immune response with a positive serology or a host who presents clinical manifestations. These manifestations are particularly important in case of immunocompromised patients. The skin might play a key role in the process of tolerance as the first interface met by the tick and the pathogen (13, 190). By modulating the host immune system, the ticks prepares the skin to the pathogen inoculation by various sophisticated mechanisms targeting all the skin cells (97, 185) The numerous molecules contained in tick saliva facilitate the process (93, 97). Although in certain circumstances as repeated infestations (318), the tick can be rejected, most of the time it remains attached to the skin. **The pathogen** uses the tick to facilitate its transmission and then multiply and persist in the skin. This has been particularly well-studied in Lyme disease (13), in mouse (314, 315) and in dog model (319). It will be very interesting to perform similar analyses in human. The exact process of tolerance at the skin interface is only starting to be defined. In VBDs, certain skin structures and cells seem to be involved in this process like adipocytes (15, 173) and the hair follicle (175).

To orchestrate these different interactions, the **microbiome** developed a sophisticated tuning. First, within the tick gut, it is the interaction of tick innate immunity with the tick microbiome, which contributes to determining the pathogenicity of the microorganisms. What makes one rickettsia a pathogen and the other one a symbiont? These practical questions remain to be answered. At the skin interface, the microbiome also contributes to regulation of inflammation and likely the host response to the tick bite. Due to the long blood feeding of a hard tick and the formation of a feeding pool, the skin microbiome of the vertebrate host enters the dermis. What is the role of the microbiota in the case of pathogens co-inoculated with tick saliva? Some preliminary data exists for certain VBDs like malaria (320) and leishmaniasis (275), but none for TBDs. In malaria, the skin microbiome is clearly involved in attractiveness of mosquito and the intestinal microbiome of the vertebrate host seems also to influence the outcome of the disease, at least in mouse model. Depletion of the bacteria from the phylum Firmicutes, mainly Gram (+) bacteria, is correlated with more severe disease (320).

Recently, **non-coding RNAs** (long non-coding RNAs and small non-coding RNAs: siRNAs, miRNAs, and piRNAs) have drawn much attention due to their diversity of function (4). It appears that small RNAs are particularly interesting because of their role in different regulation systems like innate immunity but also in the communication between host and pathogens, for example, from humans to malaria parasite and from *Escherichia coli* bacteria to *Caenorhabditis elegans* nematode (317). This phenomenon is referred to trans-kingdom silencing (317). We can then question at which level would they play a role in the different interactions occurring in TBDs and then whether they participate in the induction of tolerance. In tick saliva, several micro RNAs have been identified *in silico* that could target host genes, especially those related to inflammation (4). They also seem to be involved in the regulation of tick infection (150) and during the process of arbovirus transmission (149).

Our understanding of **skin immunity** has also made tremendous progress. The innate immunity with the engagement of PAMs with PRRs leads to secretion of cytokines, chemokines and AMPs. They chemoattract different cells to the site of tick feeding and they also activate ILCs which in turn secrete cytokines and express cell receptors that activate T cells (180). A focus for future research is the connection of the skin immune system and the nervous system (196).

To explore this complex network, molecular techniques have been very helpful. Next generation sequencing of 16S ribosomal RNA gene allowed a better identification of microbiomes (3). We need now to validate many of these observations *in situ*, in animal models or even better in patients. We require new tools in addition to molecular techniques and traditional proteomics, like targeted quantitative proteomics (321–323). It has been tested successfully on skin biopsies of mouse and human infected by *B. burgdorferi* sl to detect markers of infection (315, 324). It might be used to identify tick saliva proteins inoculated into the host skin. Targeted proteomics will be also very useful to identify vaccine candidates (325). To develop effective vaccines against VBDs, after identification of good vaccine candidates, efficient delivery system will be necessary in the future to take into consideration the skin microbiome and the skin immunity (326).

Due to the complexity of the system, involving different expertise (immunology, entomology, ecology, human and veterinary medicines, etc.), there is a real need for multidisciplinary team to answer these different scientific questions (327, 328) and find new tools to control expanding TBDs (8, 98, 329, 330).

## Author Contributions

Both authors searched, read the literature, edited the manuscript, and wrote the manuscript.

## Conflict of Interest

SW is Senior Science Advisor for U.S. Biologic, a biotechnology company that develops solutions for tick-borne diseases, pet health, and antimicrobial resistance in poultry and livestock through oral vaccines. The remaining author declares that the research was conducted in the absence of any commercial or financial relationships that could be construed as a potential conflict of interest.
